# Single-Chamber Leadless Cardiac Pacemaker in Patients Without Atrial Fibrillation: Findings From Campania Leadless Registry

**DOI:** 10.3389/fcvm.2021.781335

**Published:** 2022-01-14

**Authors:** Vincenzo Russo, Antonello D'Andrea, Stefano De Vivo, Anna Rago, Gianluca Manzo, Antonio Bocchetti, Andrea Antonio Papa, Valerio Giordano, Ernesto Ammendola, Berardo Sarubbi, Paolo Golino, Antonio D'Onofrio, Gerardo Nigro

**Affiliations:** ^1^Cardiology Unit, Department of Medical Translational Sciences, University of Campania “Luigi Vanvitelli”, Naples, Italy; ^2^Department of Cardiology, Umberto I Hospital, Nocera Inferiore, Salerno, Italy; ^3^Department of Cardiology, Monaldi Hospital, Naples, Italy

**Keywords:** leadless pacemaker, atrial fibrillation, sinus node dysfunction, atrioventricular block, effectiveness, safety, complications, syncope

## Abstract

**Introduction:**

Little is known about the clinical performance of single-chamber leadless pacemaker (LLPM) in patients without atrial fibrillation (AF) as pacing indication. The aim of this study was to describe the clinical characteristics of patients who underwent single chamber LLPM implantation at three tertiary referral centers and to compare the safety and effectiveness of the single-chamber LLPM among patients with or without AF.

**Materials and Methods:**

All the consecutive patients who underwent LLPM implantation at three referral centers were analyzed. The indications to LLPM in a real-world setting were described. The study population was divided into two groups according to AF as pacing indication. We assessed the procedure-related complications; moreover, we compared syncope, cardiac hospitalization, pacemaker syndrome, and all-cause death recurrence during the follow-up between patients with and without AF as pacing indication.

**Results:**

A total of 140 consecutive patients (mean age, 76.7 ± 11.24 years, men 64.3%) were included in the study. The indication to implantation of LLPM was permanent AF with slow ventricular response (*n*: 67; 47.8%), sinus node dysfunction (*n*: 25; 17.8%), third atrioventricular block (AVB) (*n*: 20; 14.2%), second-degree AVB (*n*: 18; 12.8%), and first degree AVB (*n*: 10; 7.1%). A total of 7 patients (5%) experienced perioperative complications with no differences between the AF vs. non-AF groups. During a mean follow-up of 606.5 ± 265.9 days, 10 patients (7.7%) died and 7 patients (5.4%) were reported for cardiac hospitalization; 5 patients (3.8%) experienced syncope; no patients showed pacemaker syndrome. No significant differences in the clinical events between the groups were shown. The Kaplan–Meier analysis for the combined endpoints did not show significant differences between the AF and non-AF groups [hazard ratio (HR): 0.94, 95% CI: 0.41–2.16; *p* = 0.88].

**Conclusion:**

Our real-world data suggest that LLPM may be considered a safe and reasonable treatment in patients without AF in need of pacing. Further studies are needed to confirm these preliminary results.

## Introduction

The leadless pacemaker (LLPM) is a miniaturized, self-contained cardiac pacemaker that emerged as a meaningful alternative to a transvenous pacemaker for single-ventricular pacing in patients at high-infectious risk or with upper limbs venous occlusion or anatomical constraints (1). Permanent atrial fibrillation (AF) with a slow ventricular rate is the most common indication for single chamber LLPM (2); however, nearly one-third of patients selected to receive this therapy were for indications not associated with AF (3). The outcome of LLPM in the real-world setting was associated with a low risk of complications and good electrical performance up to 1 year after implantation compared to a transvenous pacemaker (4). Actually, there are a few data about the clinical performance of LLPM in patients with pacing indication not associated with AF (3) and no data are still available in a real-world setting. The aim of this study was to compare the safety and effectiveness of single-chamber LLPM among patients with or without AF as a pacing indication in a real-world setting.

## Materials and Methods

The Campania Leadless Registry is an observational real-world multicenter registry that included all the consecutive patients who underwent LLPM implantation from July 2017 to December 2020 at three tertiary referral hospitals in Campania Region—Italy (Monaldi Hospital of Naples, University of Campania “*Luigi Vanvitelli*” of Naples and Umberto I Hospital of Nocera Inferiore). All the patients received Micra transcatheter pacemaker system (Medtronic, Minneapolis, Minnesota, USA) because it was the only available LLPM in Italy at the time of this study. All the procedures were performed by expert electrophysiologists who were trained at a special training laboratory with a hands-on simulator. At implantation, anthropometric, anamnestic, clinical, and intraoperative pacemaker parameters were collected. At each follow-up visit, performed at 1- and 4-weeks post-implantation and every 6 months thereafter, clinical status, pacemaker electric parameters, the occurrence of syncope, cardiac hospitalization, pacemaker syndrome, and survival were assessed. In case of missed follow-up, the patient was contacted by phone; after two unsuccessful phone contact attempts, information on the life status of the patients was collected from the regional healthcare information platform. Informed consent was obtained from all the participants before inclusion in the database. The database and this analysis were approved by the local institutional review committee (ID: 120717).

### Outcomes

The outcomes of interest were the LLPM intraoperative data, the perioperative complications, the occurrence of syncope, cardiac hospitalization, pacemaker syndrome, and all-cause death. The implant duration was defined as the time between the femoral vein cannulation and decannulation after implantation of LLPM. The perioperative complications were defined as adverse events that occurred intraoperatively or within 30 days post-operatively. The occurrence of syncope was based on self-reported data. The cardiac hospitalization and all-cause mortality were collected from the regional healthcare information platform. The pacemaker syndrome was defined as the development of either congestive signs or symptoms associated with retrograde conduction during single-chamber pacing or a ≥20 mm Hg reduction of the systolic blood pressure, associated with reproducible symptoms of weakness, lightheadedness, or syncope.

### Statistical Analysis

Categorical data were expressed as number and percentage, while continuous variables either as a median and interquartile range (IQR) or mean and SD based on their distribution assessed both by the Kolmogorov–Smirnov and the Shapiro–Wilk tests. Between the group differences, for categorical variables, were assessed by the chi-squared test, as the sample size was > 50 subjects, with the application of Yates correction where appropriate. Either the parametric Student's *t*-test or the non-parametric Mann–Whitney *U* test and Wilcoxon signed-rank test were instead used to compare continuous variables, according to their distribution. The Kaplan–Meier analysis was further performed to assess the risk of combined endpoints (syncope, cardiac hospitalization, and mortality) between the two subgroups. A two-sided probability *p* < 0.05 was considered statistically significant. All the analyses were performed using the SPSS statistical software (version 24.0, SPSS Chicago, Illinois, USA) and the STATA 14.0 software (StataCorp LLP, College Station, Texas, USA).

## Results

### Study Population

A total of 140 consecutive patients (mean age 76.7 ± 11.24 years, men 64.3%) who underwent LLPM at our referral centers were included in the study. The indication to LLPM implantation was permanent AF with slow-ventricular response (*n*: 67; 47.8%), sinus node dysfunction (*n*: 25; 17.8%), third atrioventricular block (AVB) (*n*: 20; 14.2%), second-degree AVB (*n*: 18; 12.8%), and first-degree AVB (*n*: 10; 7.1%). A total of 96 (68.1%) and 61 (43.6%) patients experienced a history of presyncope and syncope, respectively. The study cohort was further split into two subgroups based on the permanent AF as primary-pacing indication. All the baseline clinical characteristics of the study population are given in Table 1.

**Table 1:** Baseline characteristics of study population.

	**Overall population *n*: *140***	**AF group *n*: *67***	**No-AF group *n*: *73***	***P***
Age, years	76.7 ± 11.24	78.1 ± 10.8	75.5 ± 11.2	*0.16*
Male, *n* (%)	90 (64.3)	41 (61.2)	49 (67.1)	*0.47*
Hypertension, *n* (%)	98 (70)	54 (80.5)	44 (60.3)	*0.0095*
Diabetes, *n* (%)	43 (30.7)	19 (28.3)	24 (32.9)	*0.56*
COPD, *n* (%)	22 (15.7)	7 (10.4)	15 (20.5)	*0.10*
Dyslipidemia, *n* (%)	70 (50)	34 (50.7)	36 (49.3)	*0.87*
CKD, *n* (%)	28 (20)	9 (13.4)	16 (21.9)	*0.2*
Dialysis, *n* (%)	11 (7.8)	2 (3.0)	9 (12.3)	*0.0042*
Anemia, n, (%)	16 (11.4)	13 (19.4)	3 (4.1)	*0.0046*
Malignancy, *n* (%)	18 (12.8)	10 (14.9)	8 (10.9)	*0.48*
DCM, *n* (%)	30 (21.4)	14 (20.9)	16 (21.9)	*0.88*
CAD, *n* (%)	41 (29.3)	16 (23.9)	25 (34.2)	*0.18*
Pre-syncope, *n* (%)	96 (68.6)	51 (76.1)	45 (61.6)	*0.06*
Syncope, *n* (%)	61 (43.6)	19 (28.3)	42 (57.5)	*0.0005*
Infectious Leads extraction, *n* (%)	16 (11.4)	3 (4.5)	13 (17.8)	*0.0138*

*COPD, chronic obstructive pulmonary disease; CKD, chronic kidney disease; DCM, dilated cardiomyopathy; CAD, coronary artery disease*.

The non-AF group showed the lower prevalence of hypertension (60.3 vs. 80.5%; *p* = 0.009), anemia (4.1 vs. 19.4%; *p* = 0.005), and higher prevalence of patients who underwent infectious leads extraction (17.8 vs. 4.5%; *p* = 0.014) and dialysis (12.3 vs. 3%; *p* = 0.004) compared with the AF group.

### LLPM Implantation Procedure

All the patients underwent a successful implantation procedure according to the standard technique (1). The mean procedure duration time was 45.21 ± 18.59 min and the mean fluoroscopy time was 9.05 ± 6.23 min. The non-AF group showed a slightly longer procedure time compared with the AF group (49.24 ± 21.56 vs. 43.10 ± 13.23; *p* = 0.046). No differences in LLPM electrical parameters were reported between the two groups (Table 2). A total of 7 patients (5%) experienced perioperative complications with no differences between the two groups (Table 3). No procedure-related complications led to perioperative death.

**Table 2:** Intraoperative data and electrical parameters.

	**Overall population**	**AF group**	**No-AF group**	***P***
	***n*: *140***	***n*: *67***	***n*: *73***	
Implant duration, minutes	45.21 ± 18.59	43.10 ± 13.23	49.24 ± 21.56	*0.0465*
Fluoroscopy time, minutes	9.05 ± 6.23	9.09 ± 5.16	9.24 ± 7.11	*0.89*
R wave amplitude, mV	12.08 ± 4.93	11.32 ± 4.75	12.19 ± 4.84	*0.29*
Ventricular threshold, V	1.25 ± 0.75	1.45 ± 0.63	1.12 ± 1.24	*0.05*
Ventricular impedance, Ohm	792.4 ± 214.4	788.22 ± 228.78	784.58 ± 201.65	*0.92*

**Table 3:** Perioperative complications.

	**Overall population *n*: *140***	**AF group *n*: *67***	**No-AF group *n*: *73***	***P***
Pericardial effusion, *n* (%)	2 (1.4)	0 (0)	2 (2.6)	*0.19*
Inguinal hematoma, *n* (%)	1 (0.7)	1 (1.5)	0 (0)	*0.29*
Femoral Pseudoaneurysm, *n* (%)	1 (0.7)	1 (1.5)	0 (0)	*0.28*
Device dislocation, *n* (%)	1 (0.7)	0 (0)	1 (1.3)	*0.36*
High ventricular threshold, *n* (%)	2 (1.4)	1 (1.5)	1 (1.3)	*0.92*

### Follow-Up

**Figure 1:**
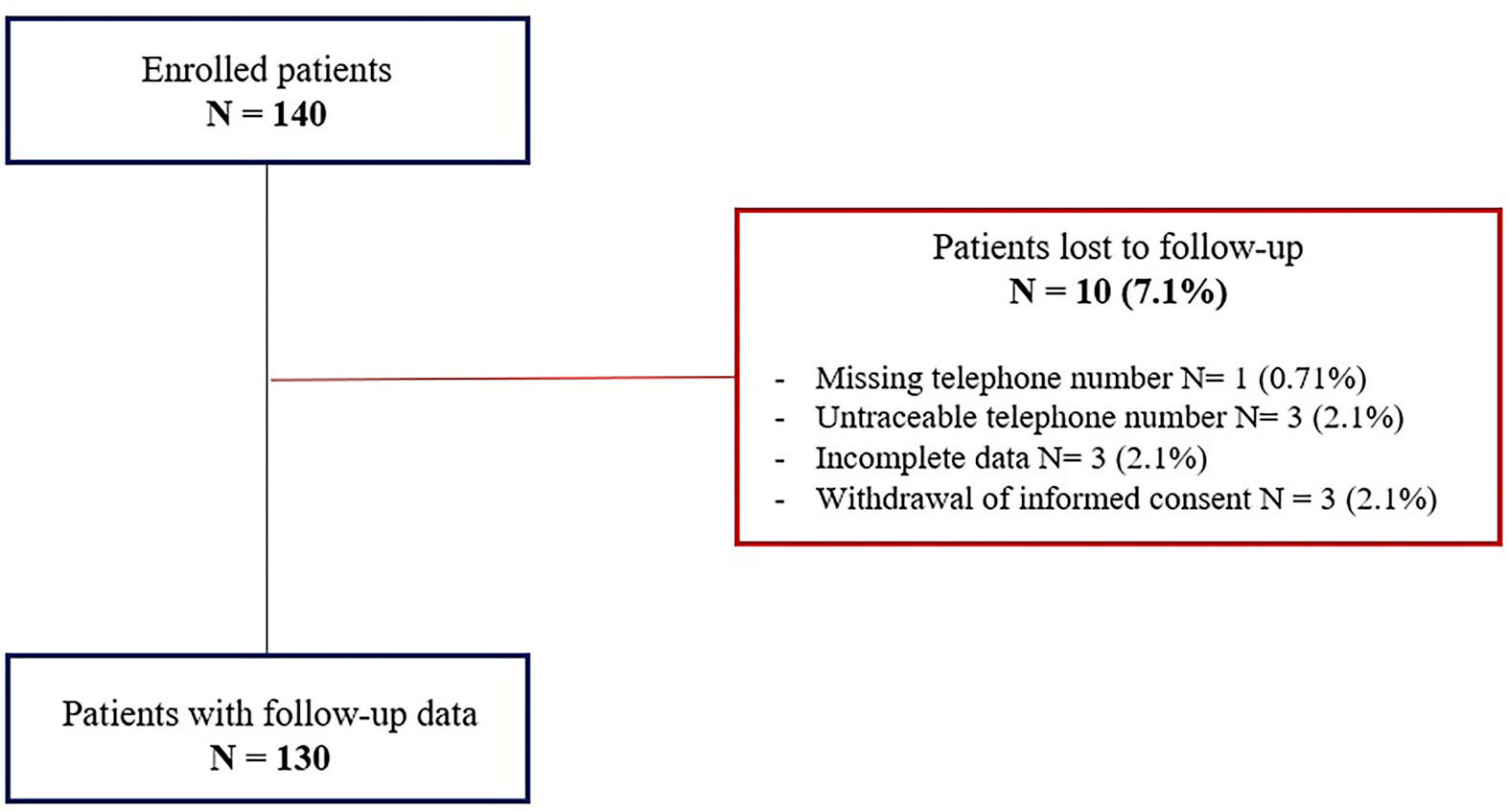
Study of the flowchart.

**Figure 2:**
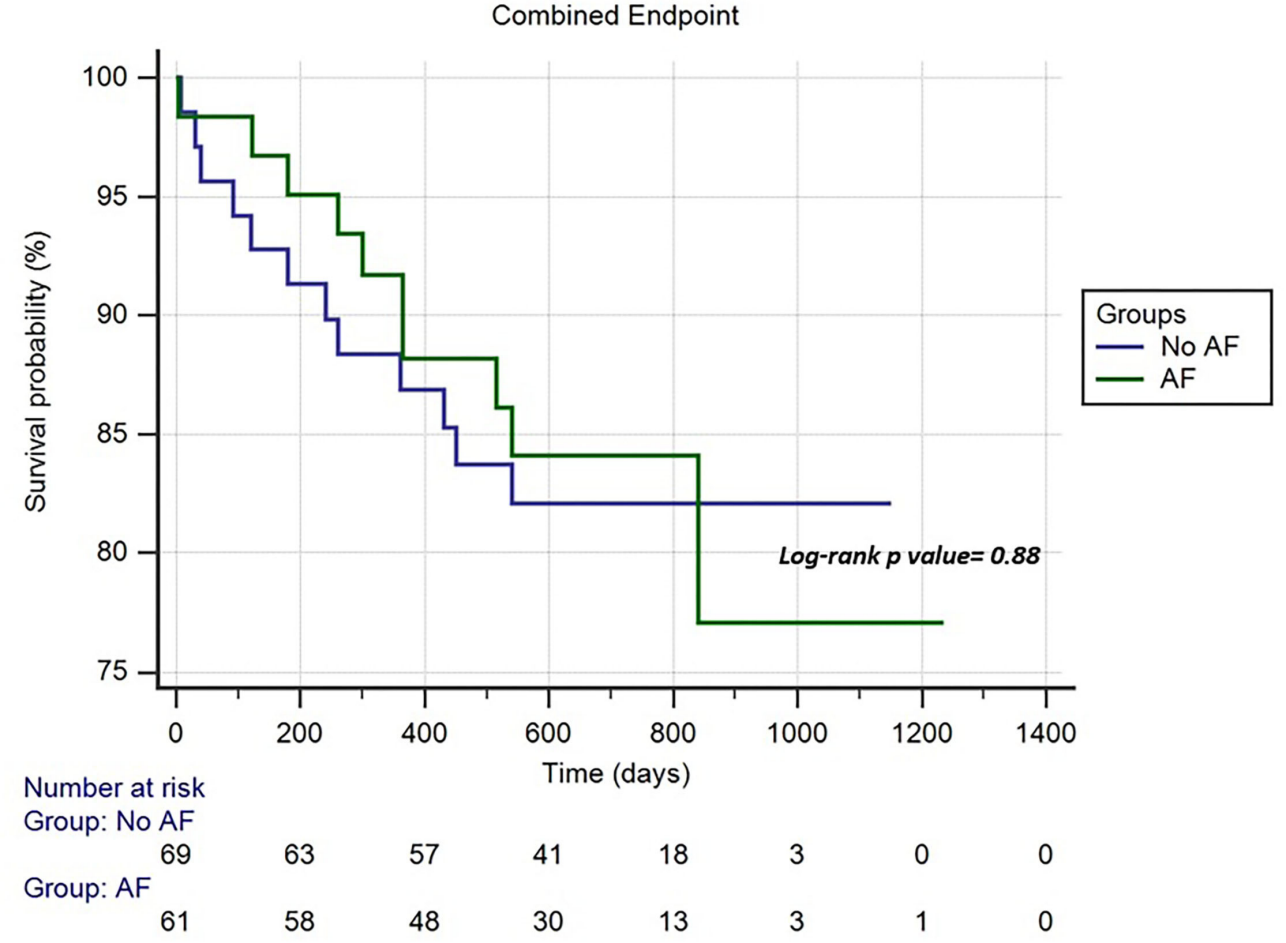
Cumulative risk of combined endpoint (syncope, cardiac hospitalization, and mortality) between the atrial fibrillation (AF) and non-AF groups.

The follow-up data were gathered to 130 patients (77.4 ± 10.9 years; 63.8% males). Figure 1 shows the study flow chart and the causes of loss to follow-up. The mean follow-up was 606.5 ± 265.9 days with no significant difference between the AF vs. non-AF groups (620.3 ± 259.1 vs. 591.1 ± 274.7 days; *p* = 0.5). The clinical characteristics and pharmacological therapies were stable over time. The pacing mode was a ventricular demand pacing (VVI) at 50 bpm in 79 patients (69.8%) and a rate responsive VVI (VVIR) at 50 bpm in 41 patients (31.5%). The non-AF group showed a higher percentage of ventricular pacing (52 ± 36 vs. 40 ± 29%; *p* = 0.002). The LLPM electrical parameters remained stable over time and did not differ between the two groups (Table 4). During the follow-up, 10 patients (7.7%) died; 7 patients (5.4%) reported cardiac hospitalization; 5 patients (3.8%) experienced syncope; no patients showed pacemaker syndrome. No significant differences in the outcome of interest were shown between the groups. The Kaplan–Meier analysis for the combined endpoints did not show significant differences between the AF and non-AF groups (HR: 0.94, 95% CI 0.41–2.16; *p* = 0.88) (Figure 2).

**Table 4:** Electrical parameters and clinical events at follow-up.

	**Overall population *n*: *130***	**AF group *n*: *61***	**No AF group *n*: *69***	***P***
**Electrical parameters**				
R wave amplitude, mV	13.75 ± 5.04	11.8 ± 5.2	10.9 ± 4.8	*0.32*
Ventricular threshold, V	1.2 ± 0.4	0.53 ± 0.45	0.55 ± 0.37	*0.79*
Ventricular impedance, Ohm	716.9 ± 187.4	707.9 ± 168	711 ± 187	*0.92*
Ventricular pacing (%)	40 ± 29	31 ± 16	52 ± 36	*0.002*
**Clinical events**				
Syncope, *n* (%)	5 (3.8)	2 (3.3)	3 (4.3)	*0.71*
Cardiac hospitalization, *n* (%)	7 (5.4)	3 (4.9)	4 (5.8)	*0.82*
All-cause death, *n* (%)	10 (7.7)	5 (8.2)	5 (7.2)	*0.83*

## Discussion

The results of our multicenter registry showed that more than half of patients with LLPM had a pacing indication not associated with permanent AF. Moreover, there was no difference in LLPM procedure-related complications, when stratified according to the primary pacing indication (AF vs. non-AF); non-AF patients who received LLPM were more likely on dialysis or following infectious leads extraction; no significant difference in syncope recurrence, cardiac hospitalization, and all-cause mortality was shown between the two groups during the follow-up.

Recently, we observed a gradual small increase in single-chamber LLPM implantation rate in patients who do not need resynchronization therapy, more likely in those presenting with AF or a high-anticipated risk of infection (5). This tendency might be explained by the fewer major complications at 1-year follow-up compared with patients with transvenous systems, mainly attributed to a lack of dislodgement and a lower rate of system revision (6, 7).

Despite the operator learning curve, we reported a low number of major intraoperative complications, in particular pericardial effusion, with no remarkable difference from those described by the Micra Transcatheter Pacing (IDE) Trial (1) and the Micra Transcatheter Pacing System Post-Approval Registry (8).

Regarding the pacing indication, 53% of our study population received single-chamber LLPM for sick sinus syndrome or AVB; the extensive use of LLPM in our clinical practice might be related to the high prevalence of risk factors for the cardiac implantable electronic device (CIED) infection among our population, such as diabetes, chronic kidney disease, malignancy, systemic anticoagulation, and prior CIED infection (9, 10).

In patients with sinus node dysfunction and AVB, dual-chamber pacing is recommended over the single-chamber pacing; however, in those in which frequent ventricular pacing is not expected or with significant comorbidities impacting on patients' outcome, single-chamber ventricular pacing is reasonable (11).

The single-chamber pacing does not impact the mortality or major cardiovascular events in elderly patients with AVB (12) or with sinus node dysfunction (13); however, it shows an increased risk of AF, and patients with higher percentages of ventricular pacing experienced an increased risk of the heart failure, regardless of pacing mode (14).

In this study, no significant difference in syncope events, cardiac hospitalizations, and all-cause mortality have been shown between LLPM patients with and without AF as primary pacing indication, despite the non-AF group showing a higher percentage of ventricular pacing.

Our real-world data confirm the evidence by Piccini et al. (3) which showed no significant difference in a composite outcome including heart failure, pacemaker syndrome, and syncope events between patients with and without AF indication or history in the IDE trial. Our findings suggest the hypothesis that, in the absence of technical issues, the LLPM could be considered a safe and reasonable treatment in patients without AF in need of cardiac pacing. This approach may constitute an option in the selected clinical settings (e.g., high risk, infectious lead extractions, etc.) in order to avoid the risks of *de-novo* dual chambers pacemaker implantation. The LLPM with automated, enhanced accelerometer-based algorithms (15) that provide atrioventricular synchronous pacing should be used for longer follow-up studies, in order to fully understand the potential clinical value of this strategy. Actually, the use of LLPM is still considerably limited by reimbursement issues and the availability of the device in many European countries.

### Limitations

Our findings might be affected by several biases. The retrospective design and the non-randomized comparison between the groups limit the strength of our results. Moreover, the small cohort size, the differences in the baseline clinical characteristics between the groups, and the limited length of follow-up limits represent the additional limitations.

## Conclusion

More than half of the patients who underwent LLPM in a real-world setting had a pacing indication not associated with permanent AF; this subgroup did not show significant differences in intraoperative major complications and terms of syncope recurrence, cardiac hospitalization, and all-cause mortality compared to those with AF. Our results suggest that LLPM may be considered a safe and reasonable treatment in patients without AF in need of pacing. Further studies are necessary to confirm our preliminary results.

## Data Availability Statement

The raw data supporting the conclusions of this article will be made available by the authors, without undue reservation.

## Ethics Statement

The studies involving human participants were reviewed and approved by Monaldi Hospital. The patients/participants provided their written informed consent to participate in this study.

## Author Contributions

VR and AD designed the study. SD, AR, GM, AP, and VG collected the data. AB, BS, and EA perfomed statistical analysis. VR, PG, and GN wrote the manuscript. All authors read and revised the manuscript.

## Conflict of Interest

The authors declare that the research was conducted in the absence of any commercial or financial relationships that could be construed as a potential conflict of interest.

## Publisher's Note

All claims expressed in this article are solely those of the authors and do not necessarily represent those of their affiliated organizations, or those of the publisher, the editors and the reviewers. Any product that may be evaluated in this article, or claim that may be made by its manufacturer, is not guaranteed or endorsed by the publisher.
